# Intravitreal Inflammation: From Benchside to Bedside 2013

**DOI:** 10.1155/2014/286571

**Published:** 2014-02-10

**Authors:** Mario R. Romano, John Christoforidis

**Affiliations:** ^1^Department of Ophthalmology, Humanitas Research Center, Rozzano, 20089 Milan, Italy; ^2^Department of Neuroscience, University of Naples Federico II, 80131 Naples, Italy; ^3^Department of Ophthalmology, College of Medicine, The University of Arizona, Tucson, AZ 85711, USA

The aim of this special issue is to describe the role of inflammation in biological mechanisms of long-known diseases, providing an up-to-date viewpoint for therapeutic approaches. The authors provide a comprehensive overview, with original and review articles, on the implications of inflammation in proliferative vitreous retinopathy, on diabetic retinopathy, and on the potential iatrogenic damage induced by sustained intravitreal anti-VEGF therapy.

Despite the copious literature, the exact mechanism of idiopathic epiretinal membranes formation (iERM) is still unclear. M. Joshi et al. showed the importance of glial cells and hyalocytes in its pathogenesis. Such cellular constituents of iERMs in concert with cytokines, growth factors (TGF and NGF), and anomalous posterior vitreous detachment are responsible for the prognosis and potential growth of epiretinal traction. The proliferative vitreoretinopathy (PVR) is recognized as a common secondary severe inflammatory complication of retinal detachment. C. Azzolini et al. reported two possible pathways (VEGF-A, Otx2, p53, p63, and Otx1 and Otx3) of PVR. These genetic pathways could represent a novel target of therapy of PVR progression. PVR is an even more common complication in patients with open-globe injury. F. Morescalchi et al. describe the role of immune cells into the vitreous gel in penetrating ocular injury. The cells stimulate the production of growth factors and cytokines, in particular PDGFR-*α*, which seem to be crucial in the development of PVR and, therefore, considered another potential therapeutic target. In a prospective, nonrandomized, observational study, C. Costagliola et al. showed that tear fluid collection is a useful and noninvasive method for the finding of proliferative diabetic retinopathy. The authors reported that TNF-alpha concentrations increase in tears according to the severity of proliferative and nonproliferative diabetic retinopathy.

F. Parmeggiani et al. demonstrated the evidence of a relationship between AMD-risk genotypes, immunoinflammatory endophenotypes, and the networks of acquired or epigenetic factors.

The authors reported that proinflammatory effects secondary to chronic inflammation and heterogeneous types of oxidative stress induce degenerative damage to the photoreceptors and outer retinal-blood barrier. In particular, the carriers of CFH, ARMS2/HRTA1, and C2/CFB genotypes demonstrated high odd ratio values. A score, calculating a risk including genetic findings, could be useful to identify individual risk, in order to provide preventive treatments.

The intraviteral inflammation can be also secondary to iatrogenic treatment as intravitreal anti-VEGF therapy or vitreoretinal surgery.

S. Agrawal et al. compared the sterile inflammation among the different intravitreal anti-VEGF drugs currently used for neovascular diseases. The acute intraocular inflammation is more frequently associated with bevacizumab likely due to the less strict purification procedure of the drug. In most cases, the inflammation solves spontaneously and vision returns to baseline. A history of previous inflammation does not increase the risk with following intravitreal injections.

Previous vitreoretinal surgery can also induce intravitreal inflammation responsible for cytotoxic macular oedema. V. Romano et al. reported that intravitreal inflammation may increase after pars plana vitrectomy due to tractional phenomena at vitreomacular interfaces or due to a vasogenic damage. The oedema is characterized by fluid accumulation in the parenchymatous retinal cells (intracellular oedema) or by extracellular fluid accumulation due to a blood-retinal barrier damage (extracellular oedema).

The treatment of vitreous inflammation is still a challenging issue; however, there are multiple methods of systemic treatment including T-cell inhibitors/calcineurin, antimetabolites, corticosteroids, alkylating agents, inhibitors, and biologic agents. A. Jiang et al. showed that these drugs can be used either alone or together in order to control vitreous inflammation due to variety of etiologies, either infectious or noninfectious. A. Russo et al. demonstrated that the topical nonsteroidal anti-inflammatory drugs (NSAIDs) allow for greater drug penetration into the vitreous. Topical NSAIDs reduce the exudation secondary to age-related macular degeneration or diabetic maculopathy. NSAIDs are also considered a favorable adjunct together with anti-VEGF that could potentially reduce the number of anti-VEGF injections. Finally, the chronicity of certain inflammatory conditions limits the efficacy of locally administered drugs. J. Wang et al. reported the last updates on implantable devices and particulate delivery systems such as nanoparticles, microparticles, and liposomes. These topics are actually considered the research focus of biomedical engineering, pharmacology, and molecular biology.

We wish that this special issue will provide helpful information to recognize the clinical features of vitreous inflammation, to understand the mechanism beyond, and to identify the latest treatments in the disease in which the vitreous is involved.

*Mario R. Romano* *Mario R. Romano* *John Christoforidis* *John Christoforidis*

